# Preoperative Risk Assessment of Lymph Node Metastasis in cT1 Lung Cancer: A Retrospective Study from Eastern China

**DOI:** 10.1155/2019/6263249

**Published:** 2019-12-01

**Authors:** Chengyan Zhang, Guanchao Pang, Chengxi Ma, Jingni Wu, Pingli Wang, Kai Wang

**Affiliations:** Department of Respiratory and Critical Care Medicine, Second Affiliated Hospital, Zhejiang University School of Medicine, 310009 Hangzhou, China

## Abstract

**Background:**

Lymph node status of clinical T1 (diameter ≤ 3 cm) lung cancer largely affects the treatment strategies in the clinic. In order to assess lymph node status before operation, we aim to develop a noninvasive predictive model using preoperative clinical information.

**Methods:**

We retrospectively reviewed 924 patients (development group) and 380 patients (validation group) of clinical T1 lung cancer. Univariate analysis followed by polytomous logistic regression was performed to estimate different risk factors of lymph node metastasis between N1 and N2 diseases. A predictive model of N2 metastasis was established with dichotomous logistic regression, externally validated and compared with previous models.

**Results:**

Consolidation size and clinical N stage based on CT were two common independent risk factors for both N1 and N2 metastases, with different odds ratios. For N2 metastasis, we identified five independent predictors by dichotomous logistic regression: peripheral location, larger consolidation size, lymph node enlargement on CT, no smoking history, and higher levels of serum CEA. The model showed good calibration and discrimination ability in the development data, with the reasonable Hosmer-Lemeshow test (*p* = 0.839) and the area under the ROC being 0.931 (95% CI: 0.906-0.955). When externally validated, the model showed a great negative predictive value of 97.6% and the AUC of our model was better than other models.

**Conclusion:**

In this study, we analyzed risk factors for both N1 and N2 metastases and built a predictive model to evaluate possibilities of N2 metastasis of clinical T1 lung cancers before the surgery. Our model will help to select patients with low probability of N2 metastasis and assist in clinical decision to further management.

## 1. Introduction

Preoperative staging of patients with malignant lung cancer suggests the prognosis and the life quality afterwards. An accurate clinical staging can guide physicians to choose a proper treatment according to the authorized guideline and therefore standardizes the management procedure. Especially for those with positive mediastinal lymph nodes (N2 disease), preoperative chemotherapy is reported to reduce tumor size by 25% [1], downstage nearly half of the N2-positive patients [2–6], and increase the 5-year survival rate of 5-20% compared with surgery alone [7–11]. In that case, the accuracy of TNM staging before surgery is of paramount important.

The European Society of Thoracic Surgeons (ESTS) guidelines compared the diagnostic accuracy of different preoperative examinations for lymph node evaluation. Computed tomography is common and available in most countries, despite its low sensitivity (55%) and specificity (81%) [12, 13]. PET-CT scan is reported to be superior to CT in mediastinal lymph node staging and exhibits a high negative predictive value (NPV) for peripheral tumors. The sensitivity of PET-CT is 80-90%, and the specificity is 85-95% [12, 13]. However, PET-CT requires more expensive facilities and is not as popularized as CT. Besides, the negative predictive value of PET-CT decreases in patients with central tumors, tumors > 3 cm, and suspected N1 metastasis [12].

Reported data shows the prevalence of occult N2 disease in patients with clinical stage I NSCLC is about 5.0-6.5% [14, 15]. In order not to omit this part of patients, a predictive model in combination of assisted examination is needed and previous efforts have been made by researchers. In this study, we aim to analyze the clinical features of patients with lymph node metastasis and create a predicted formula of N2 metastasis for clinical T1 lung cancers.

## 2. Methods

### 2.1. Patients

**Figure 1:**
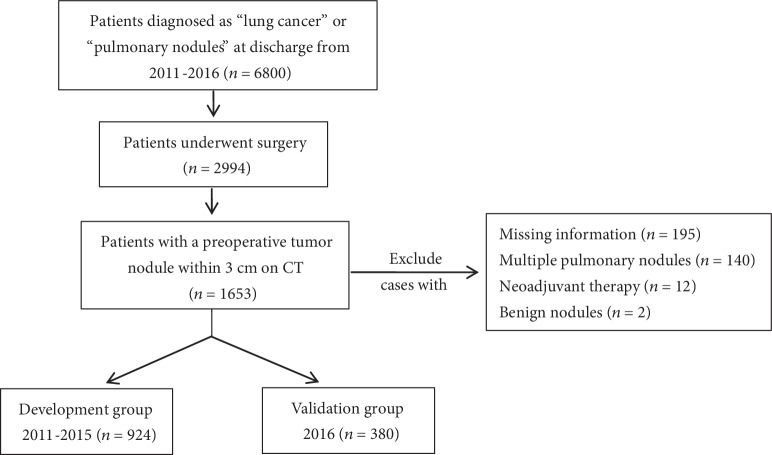
Flowchart of patient selection and exclusion.

We retrospectively reviewed patients who were diagnosed with lung cancer and underwent radial surgical recession in Second Affiliated Hospital of Zhejiang University (SAHZU) during 2011-2016. Patients with a malignant nodule within 3 centimeters on CT (staged as cT1) were selected, all of which underwent lymph node evaluation via surgical operation. The exclusion criteria were as follows: (1) patients with multiple pulmonary cancers or metastatic pulmonary nodules, (2) patients with a history of preoperative therapy, and (3) patients without CT scan images before surgery. Patients from 2011 to 2015 were enrolled in the development group (*n* = 924), while patients from 2016 were included in the validation group (*n* = 380), as shown in Figure 1. This study was approved by the Institutional Ethics of Committee of SAHZU (2017-031).

### 2.2. Clinicopathological Variables

All the clinicopathological information was collected in the hospital information system (HIS). Information included gender, age, symptoms at presentation, smoking history, smoking index, chronic pulmonary diseases, cancer history, family history of cancer, levels of tumor markers within one month before surgery, histological type of lung cancer, pathological report of resected lymph nodes, tumor location (upper/middle/lower lobe, central/peripheral location), tumor size, consolidation size, C/T ratio (consolidation size/tumor size), and clinical N stage based on CT. Chronic pulmonary diseases included chronic bronchitis, emphysema, and chronic obstructive pulmonary disease (COPD). Tumor size was measured as the largest dimension on CT section in pulmonary window while consolidation size was measured in mediastinal window. Tumors were defined as peripherally located if the center of tumor mass was in the outer one-thirds of pulmonary parenchyma and otherwise as centrally located. A lymph node was considered an enlarged one when its short axis exceeded 1 cm. The seventh edition of TNM classification was referred to in this study.

### 2.3. Data Analysis

All the continuous variables were described with means and standard deviations, while categorical variables were described with frequencies. In univariate analysis, we performed one-way analysis of variance for continuous variables and Pearson's chi-square tests (adjusted *p* values using Bonferroni method) for categorical variables. Significant variables in the univariate analysis were further analyzed in multivariate analysis using polytomous logistic regression, in order to estimate different risk factors and odds ratios for each N stage (pN0, pN1, and pN2).

The dichotomous logistic regression was performed to build a predictive model for N2 metastasis, since N2 metastasis is worse in TNM staging and requires different preoperative treatment strategies. All variables collected from HIS were analyzed with forward stepwise selection, which was based on statistics of a conditional likelihood ratio test. A significant *p* value for entering variables was 0.05, and the *p* value for excluding variables was 0.10. The optimal cutoff point of the model was set according to the highest Youden's index. A nomogram was developed using the package of rms based on the logistic regression. In addition, calibration of the model was established with the Hosmer-Lemeshow goodness-of-fit test as well as the calibration curve, and the discrimination ability of the model was assessed by receiver operating characteristic (ROC) analysis. The DeLong test was performed for the comparison of different ROC curves.

All statistical analysis was performed using SPSS Statistics 22.0 (IBM Armonk, NY, USA), EmpowerStats software (X&Y Solutions, Boston, USA, http://www.empowerstats.com/), and R 3.5.2 software (R Foundation for Statistical Computing, Vienna, Austria). We considered the differences as statistically significant when two-sided *p* values were less than 0.05.

## 3. Results

### 3.1. Clinicopathological Characteristics for Patients in the Development Group

The clinicopathological characteristics of 924 patients in the development group are shown in Table 1. Patients were at a mean age of 59.1 ± 9.7, and tumor sizes were 1.70 ± 0.62 cm on average. The incidence for lymph node metastasis was 10.82% (100/924), with N1 metastasis being 3.24% (30/924) and N2 metastasis being 7.58% (70/924).

In univariate analysis (Table 1), lymph node metastasis was prone to be found in smoking males who suffered from chronic pulmonary diseases and were hospitalized with respiratory- or cancer-related symptoms (RCRS) and higher levels of carcinoembryonic antigen (CEA). Tumors with larger size (or consolidation size), central location, and lymph node enlargement on CT images were associated with higher likelihood of lymph node metastasis. Besides, patients with squamous carcinoma were more likely to have N1 metastasis, while N2 metastasis in patients with adenocarcinoma was three times more likely to occur than N1 metastasis.

### 3.2. Odds Ratios of N1 and N2 Metastases versus N0 Status

In polytomous logistic regression (Table 2), significant variables in univariate analysis were further analyzed to estimate the risk factors and odds ratios of nodal metastasis stratified by the 7^th^ TNM staging. Significantly elevated odds ratios were seen in tumors with larger consolidation size and lymph node enlargement on CT for N1 metastasis (OR_consolidation size_ = 5.449, 95% CI: 2.817-10.541; OR_lymph node enlargement on CT_ = 11.424, 95% CI: 3.316-39.360) and N2 metastasis (OR_consolidation size_ = 8.640, 95% CI: 5.002-14.923; OR_lymph node enlargement on CT_ = 8.703, 95% CI: 4.326-17.509) compared to N0 status. A significantly decreased odds ratio was seen in smokers for N2 metastasis (OR_smoking history_ = 0.217, 95% CI: 0.080-0.590) compared to N0 status in nonsmokers. Tumors with a central location seemed to have a negative correlation with N2 metastasis though there was no significant difference.

### 3.3. Logistic Regression Model and Predictors of N2 Metastasis

**Figure 2:**
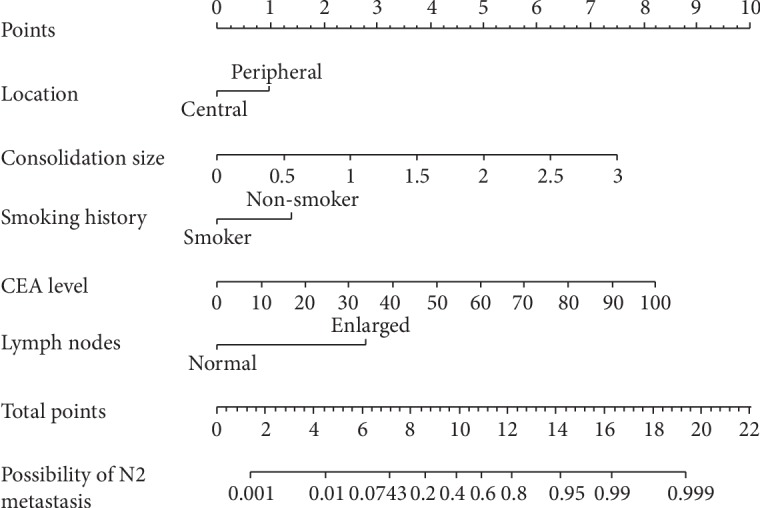
Nomogram predicting the likelihood of N2 metastasis in early lung cancers (tumor ≤ 3 cm). According to the location of value from the 2nd to the 6th axis, we can get the vertically corresponding points on the first axis. By summing up each points, we get a total point, and the vertically corresponding predicted value on the last axis shows the predicted possibility of N2 metastasis.

Dichotomous logistic regression identified five independent predictors for N2 metastasis: peripheral location, consolidation size, lymph node enlargement on CT, no smoking history, and levels of serum CEA (Table 3). Gender, histological type, and C/T ratio were not involved as significant factors. The formula predicting N2 metastasis for small tumor nodules was established: *e*^*x*^/(1 + *e*^*x*^), *x* = −0.756^×^central location + 1.921^×^consolidation size + 2.145^×^lymph node enlargement on CT − 1.065^×^smoking history + 0.064^×^CEA level − 6.165. The unit for “consolidation size” is cm and for “CEA level” is ng/ml. The value of “lymph node enlargement on CT,” “central location,” and “smoking history” should be 1 for yes and otherwise 0. A nomogram predicting the probability for N2 metastasis in cT1 patients was developed on the basis of multivariate logistic analysis (Figure 2).

**Figure 3:**
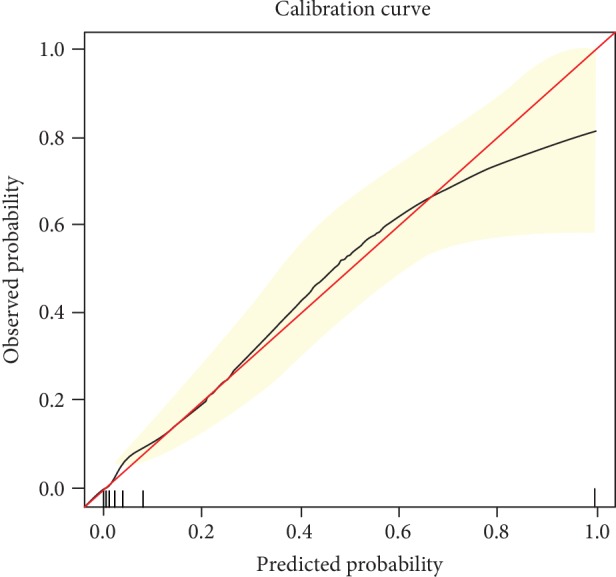
Calibration curve of the logistic regression model. The red line indicated a perfect prediction of observed possibilities. The black line represented the entire development group (*n* = 924).

**Figure 4:**
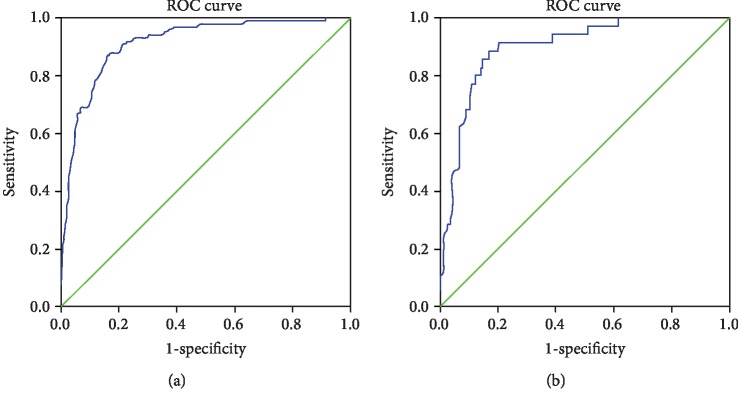
The receiver operating characteristic curve for the development and validation groups. (a) The ROC curve for the development group. The AUC was 0.931 (95% CI: 0.906-0.955). (b) The ROC curve for the validation group. The AUC was 0.906 (95% CI: 0.857-0.956).

The Hosmer-Lemeshow goodness-of-fit test, which was not statistically significant (*p* = 0.839), indicated that the predicted probability was of high concordance to the observed probability. A calibration curve is shown in Figure 3. The area under the receiver operating characteristic curve was 0.931, with 95% confidence interval between 0.906 and 0.955 (Figure 4(a)). We selected the numerical value with the highest Youden's index as our cutoff point for the predicted probability (cutoff for probability = 7.43%).

### 3.4. Validation of the Model and Comparison with Previous Models

The characteristics of patients in the validation group were shown in Supplementary [Supplementary-material supplementary-material-1]. In the external validation, the AUC of our model was 0.906 (95% CI: 0.857-0.956, Figure 4(b)). With the cutoff point set above (cutoff = 7.43%), we tested our model in the validation group. The sensitivity and specificity were 60.0% and 90.3%, respectively. The negative and positive predictive values (NPV and PPV) were 97.6% and 25.5%, respectively. In a subgroup analysis of adenocarcinoma (ADC) and squamous cell carcinoma (SCC), the validated AUC of ADC patients was 0.856 (95% CI: 0.790-0.922) and the validated AUC of SCC patients was 0.864 (95% CI: 0.777-0.952) (*p* = 0.885, DeLong test).

**Figure 5:**
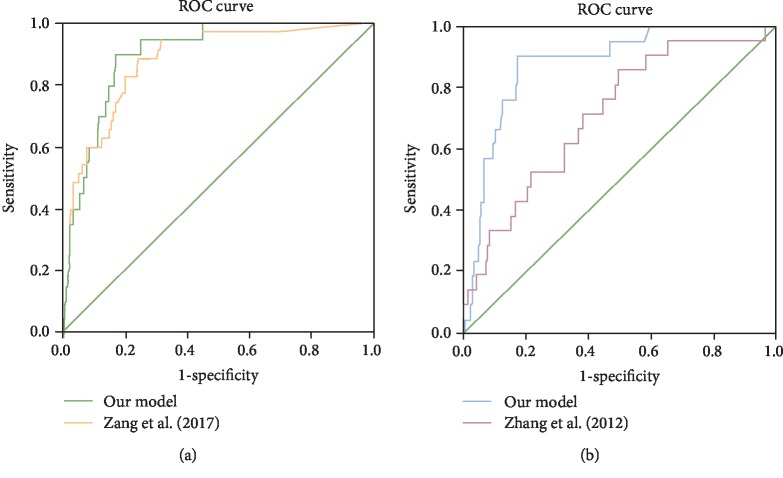
Comparison of our model and other published models using data from the same validation group. (a) Comparison with Zang et al. (2017) in cT1NxM0 patients. The AUC was 0.879 validated by our data (95% CI: 0.821-0.937). DeLong test for comparing two ROC curves: *p* = 0.405. (b) Comparison of our model with Zhang et al. (2012) in cT1N0M0 patients. The AUC was 0.712 validated by our data (95% CI: 0.602-0.822). DeLong test for comparing two ROC curves: *p* = 0.002.

We also compared our model with the Fudan model [16] and Beijing model [17], as all three studies included clinical T1 NSCLC. Analyzed with all the data from our validation group, the validated AUC of the Beijing model was 0.879 (95% CI: 0.821-0.937) compared with 0.906 (95% CI: 0.857-0.956) of our model (*p* = 0.405, DeLong test). Based on the inclusion criteria of the Fudan model, patients with cT1N0M0 lung cancers were selected from the validation group of our study. And the validated AUC of the Fudan model was 0.712 (95% CI: 0.602-0.822) while the AUC of our model was 0.885 (95% CI: 0.820-0.949) (*p* = 0.002, DeLong test). Our model showed a larger area under the ROC curve compared to other models (Figure 5).

## 4. Discussion

Lymph node status, especially the assessment of N2 metastasis, largely affects the treatment strategies in the clinic. Therefore, it is of great significance to make an accurate and noninvasive assessment of lymph nodes before operation. In this study, we established a five-variable formula predicting N2 metastasis for malignant nodules within 3 cm. Our model showed a high negative predictive value of 97.6% and specificity of 90.3%, which can select patients with low risks of N2 metastasis and help with the clinical decision-making.

As a truly multidisciplinary process, preoperative evaluation of lymph node evaluation has confused clinical physicians for many years. An algorithm that integrates imaging, endoscopic, and surgical techniques recommended by ESTS guidelines has been widely practiced and prospectively validated, with the negative predictive value as high as 0.94 [18]. However, some researchers are more interested in creating a predictive model ahead of biopsy strategy [16, 17, 19–21], because the accuracy of preoperative invasive staging such as TBNA may largely depend on the experience of operators.

Shafazand and Gould reported the first quantitative model to pretest the probability for N2 metastasis in NSCLC of all stages [20]. The formula consisted of six independent predictors, which were age, tumor size, central location, adenocarcinoma histology, onset of primary symptoms, and abnormal mediastinum on chest X-ray. However, their data was directly collected from a previous randomized controlled trial and no CT images were included at that time. After that, Zhang and colleagues reported a four-predictor model for N2 metastasis in CT-defined T1N0M0 NSCLC in 2012 [16]. Younger patients with a central-located and larger-sized lung adenocarcinoma had higher risks of N2 disease. However, patients with a histology of AIS (adenocarcinoma in situ) and MIA (microinvasive adenocarcinoma) were excluded from their study, despite the fact that the pathology of AIS or MIA could only be confirmed from a resected specimen. In that case, the percentage of adenocarcinoma might be underestimated in their model because there will be AIS and MIA patients in reality. More recently, there were predictive models evaluating N2 metastasis for NSCLC of all stages [21] and models estimating nodal metastasis in clinical T1a stages [17].

No models above have referred to the different risk factors of N1 and N2 metastases. Analyzed by polytomous logistic regression, we found that consolidation tumor size and lymph node enlargement on CT scan were the most related factors to both N1 and N2 metastases in patients with early malignant nodules (diameter ≤ 3 cm, stage T1). Though it is difficult to differentiate benign lymphadenectasis from lymph node metastasis on CT, our results showed that lymphadenectasis in N1 station was of higher correlation to N2 metastasis. This could be explained by the lymphatic drainage, and the rate for skip N2 metastasis was only 29% [22]. This result was partly in accordance with the previous literature and the ESTS recommendation [13, 23].

In both polytomous and dichotomous logistic analyses, consolidation tumor size and lymph node enlargement on CT and CEA levels were correlated to N2 metastasis, which is consistent with previous studies [16, 17, 24, 25]. Smoking history seemed to be negatively associated with N2 disease, as the odds ratio was less than 1 in both analyses. Despite the lack of molecular mechanisms, nonsmokers are more prone to a delayed or incidental detection of lung cancer than smokers and thus are more likely to progress into nodal metastasis, as supported by data from Lee et al. [26]. Apart from that, tumors with peripheral location were found with a higher likelihood of N2 metastasis in this study. The inconsistency between different research studies [27, 28] could result from the different criteria of the definition as “central location” and the different target population. Takeda et al. also found that peripheral tumors are more likely to have N2 metastasis by subpleural lymph drainage pathways [29].

Compared with previous logistic analysis, this study exhibited a larger sample size and reduced selective bias by enrolling patients with all pathological type including AIS and MIA, which constituted 8.2% and 16.7% of ground-glass nodules in the development group. Our data suggested that consolidation size was a stronger predictive factor of nodal metastasis compared with tumor size and C/T ratio in the multivariate analysis. Squamous cell carcinoma also fit in with this model though it was a minority type of histology. Besides, pathological type was not an independent factor in this multivariate model, suggesting that preoperative histology might not be a necessity for predicting N2 metastasis.

Nevertheless, this study also had several limitations. Firstly, it was a retrospective study and there was no standard on the number of resected lymph nodes. In 2014, American College of Surgeons Commission on Cancer recommended at least 10 regional lymph nodes to be removed and pathologically examined for resectable NSCLC [30]. Thus, a diagnostic bias might occur in our study. Secondly, we only collected data from a single-center institution and reflected patient characteristics in local areas. Finally, in order to ensure the general use of the model, the proportion of lymph node metastasis in this study was coherent with the prevalence in reality, which was insufficient and influenced the positive predictive value of the model. Therefore, a larger-sized study with more positive data from multiple medical centers will be needed to carry out a more practical model for clinical use.

## 5. Conclusions

In this study, we analyzed the clinical features of patients with lymph node metastasis and produced a model predicting the possibility of N2 nodal metastasis for early lung cancers (tumor ≤ 3 cm). Stratified by the cutoff point, a low predicted probability may suggest an operation directly without neoadjuvant therapies, while a relatively high predicted probability needs support from further invasive and expensive examinations. Our model will provide some clues for clinical decision-making.

## Figures and Tables

**Table 1:** Characteristics of patients in the development group.

	Patients with negative LNs (%)	Patients with positive N1 nodes (%)	Patients with positive N2 nodes (%)	*p* value^∗^
Age (year)	59.1 ± 9.7	58.1 ± 10.0	58.9 ± 10.4	0.742
Gender				
Male	346 (85.4)	21 (5.2)	38 (9.4)	0.011
Female	478 (92.1)	9 (1.7)	32 (6.2)	
Symptoms				
RCE	456 (91.9)	8 (1.6)	32 (6.5)	0.010
RCRS	256 (85.6)	16 (5.4)	27 (9.0)	
ICD	112 (86.8)	6 (4.7)	11 (8.5)	
Asymptomatic	568 (90.9)	14 (2.2)	43 (6.9)	0.008
Symptomatic	256 (85.6)	16 (5.4)	27 (9.0)	
Cancer history				
Yes	58 (85.3)	4 (5.9)	6 (8.8)	0.454
No	766 (89.5)	26 (3.0)	64 (7.5)	
Family history of cancer				
Yes	122 (91.0)	1 (0.8)	11 (8.2)	0.169
No	702 (88.9)	29 (3.7)	59 (7.4)	
Pathology				
Adenocarcinoma	760 (91.2)	16 (1.9)	57 (6.9)	<0.001
Squamous	52 (75.4)	10 (14.5)	7 (10.1)	
Adenosquamous	3 (37.5)	1 (12.5)	4 (50.0)	
Neuroendocrine	8 (66.7)	3 (25.0)	1 (8.3)	
Other tumor type	1 (50.0)	0 (0.0)	1 (50.0)	
Smoking history				
Yes	220 (85.3)	17 (6.6)	21 (8.1)	0.001
No	604 (90.7)	13 (2.0)	49 (7.3)	
Location				
Upper lobe	445 (90.1)	14 (2.8)	35 (7.1)	0.670
Lower lobe	270 (88.8)	9 (3.0)	25 (8.2)	
Middle lobe	109 (86.5)	7 (5.6)	10 (7.9)	
Central	316 (86.6)	18 (4.9)	31 (8.5)	0.042
Peripheral	508 (90.9)	12 (2.1)	39 (7.0)	
Nodule size on CT				
Tumor size (cm)	1.63 ± 0.59	2.25 ± 0.57	2.33 ± 0.50	<0.001
Consolidation size (cm)	0.91 ± 0.83	2.19 ± 0.69	2.22 ± 0.59	<0.001
C/T ratio	0.51 ± 0.41	0.95 ± 0.19	0.95 ± 0.15	<0.001
Chronic pulmonary disease				
Yes	68 (78.2)	8 (9.2)	11 (12.6)	0.001
No	756 (90.3)	22 (2.6)	59 (7.1)	
Clinical nodal stage on CT				
Enlarged LNs in N2 station	72 (64.3)	7 (6.3)	33 (29.4)	<0.001
Enlarged LNs in N1 station	10 (38.5)	6 (23.0)	10 (38.5)	
Normal-sized LNs	742 (94.4)	17 (2.2)	27 (3.4)	
Levels of tumor markers				
CEA (ng/ml)	3.26 ± 4.84	3.55 ± 2.56	10.21 ± 17.58	<0.001
AFP (ng/ml)	3.03 ± 1.80	2.68 ± 0.89	3.25 ± 2.71	0.542
CA199 (U/ml)	10.48 ± 15.24	9.99 ± 9.26	15.67 ± 17.16	0.013
CA125 (U/ml)	11.31 ± 11.22	13.23 ± 10.04	26.53 ± 77.33	0.093
CA242 (U/ml)	5.42 ± 4.34	4.80 ± 3.22	5.54 ± 3.31	0.996
CA211 (ng/ml)	1.12 ± 0.84	1.49 ± 1.07	1.36 ± 1.01	0.006
NSE (ng/ml)	9.58 ± 4.55	8.48 ± 4.87	9.99 ± 5.32	0.793
SCC (ng/ml)	0.84 ± 0.83	1.15 ± 0.78	0.93 ± 0.59	0.143

RCE: routine chest examination; RCRS: respiratory- or cancer-related symptoms; ICD: incidental chest discovery; C/T ratio: consolidation size/tumor size ratio. ^∗^*p* value acquired from one-way analysis of variance and Pearson's chi-square tests.

**Table 2:** Odds ratios of likelihood of lymph node metastasis stratified by seventh TNM staging using polytomous logistic regression.

Variable	N1 metastasis (*n* = 30)		N2 metastasis (*n* = 70)	
	Odds ratio (95% CI)	*p* value^#^	Odds ratio (95% CI)	*p* value^#^
Male gender	2.366 (0.726-7.707)	0.153	2.019 (0.868-4.697)	0.103
Chronic pulmonary disease	1.827 (0.665-5.021)	0.242	1.231 (0.489-3.102)	0.659
Smoking history	0.479 (0.138-1.663)	0.246	0.217 (0.080-0.590)	0.003
Respiratory- or cancer-related symptoms	1.558 (0.692-3.508)	0.284	0.741 (0.374-1.467)	0.389
Adenocarcinoma histology	1.742 (0.619-4.901)	0.293	0.849 (0.330-2.181)	0.733
Consolidation size (cm)	5.449 (2.817-10.541)	<0.001	8.640 (5.002-14.923)	<0.001
Central location	1.069 (0.448-2.547)	0.881	0.508 (0.255-1.014)	0.055
Clinical nodal stage on CT				
Enlarged LNs in N1 station	11.424 (3.316-39.360)	<0.001	14.046 (4.226-46.682)	<0.001
Enlarged LNs in N2 station	1.615 (0.582-4.480)	0.357	8.703 (4.326-17.509)	<0.001
Levels of serum CEA (ng/ml)	0.932 (0.806-1.077)	0.340	1.063 (1.027-1.099)	0.001

^#^ *p* value represented the comparison with N0 patients.

**Table 3:** Multivariate dichotomous logistic regression of the development group for predicting N2 metastasis.

Variable	Regression coefficient	*p* value	Odds ratio	95% confidence interval	
				Lower	Upper
Central location	-0.756	0.029	0.469	0.239	0.924
Consolidation size (cm)	1.921	<0.001	6.824	4.095	11.373
Enlarged lymph node on CT	2.145	<0.001	8.546	4.491	16.262
Smoking history	-1.065	0.003	0.345	0.169	0.704
Level of serum CEA (ng/ml)	0.064	<0.001	1.066	1.031	1.102

## Data Availability

All relevant data are within the article and the supplementary materials.

## Supplementary Materials

Supplementary Materials Supplementary Table 1: characteristics of patients in the validation group.
