# Supplementary material for: Preoperative Risk Assessment of Lymph Node Metastasis in cT1 Lung Cancer: A Retrospective Study from Eastern China
Source: J Immunol Res. 2019 Dec 1;2019:6263249. doi: 10.1155/2019/6263249 (PMC6914921; doi:10.1155/2019/6263249)
Supplement: Supplementary Materials — Supplementary Table 1: characteristics of patients in the validation group. [file 6263249.f1.doc]

## Supplementary Materials

Supplementary Table 1 Characteristics of patients in the validation group

|  |  | All patients  （n=380） | Patients with negative LNs  （n=345） | Patients with positive N1 nodes  （n=15） | Patients with positive N2 nodes  （n=20） | P value* |
| --- | --- | --- | --- | --- | --- | --- |
| Age(year) |  | 58.2±10.4 | 58.2±10.4 | 56.6±12.5 | 59.6±9.0 | 0.759 |
| Gender | male | 161 | 135 | 8 | 18 | ＜0.001 |
|  | female | 219 | 210 | 7 | 2 |  |
| Symptoms | RCE | 204 | 192 | 5 | 7 | 0.054 |
|  | RCRS | 99 | 84 | 8 | 7 |  |
|  | ICD | 77 | 69 | 2 | 6 |  |
|  | asymptomatic | 281 | 261 | 7 | 13 | 0.028 |
|  | symptomatic | 99 | 84 | 8 | 7 |  |
| Cancer history | yes | 32 | 31 | 1 | 0 | 0.471 |
|  | no | 348 | 314 | 14 | 20 |  |
| Family history | yes | 54 | 51 | 2 | 1 | 0.474 |
| of cancer | no | 326 | 294 | 13 | 19 |  |
| Pathology | adenocarcimona | 345 | 322 | 10 | 13 | <0.001 |
|  | squamous | 25 | 17 | 4 | 4 |  |
|  | adenosquamous | 3 | 1 | 0 | 2 |  |
|  | neuroendocrine | 3 | 2 | 0 | 1 |  |
|  | other tumor type | 4 | 3 | 1 | 0 |  |
| Smoking history | yes | 123 | 103 | 6 | 14 | 0.001 |
|  | no | 257 | 242 | 9 | 6 |  |
| Location | upper lobe | 204 | 189 | 8 | 7 | 0.134 |
|  | lower lobe | 130 | 112 | 6 | 12 |  |
|  | middle lobe | 46 | 44 | 1 | 1 |  |
|  | central | 176 | 151 | 11 | 14 | 0.007 |
|  | peripheral | 204 | 194 | 4 | 6 |  |
| Nodule size | tumor size(cm) | 1.54±0.64 | 1.49±0.61 | 2.14±0.61 | 1.95±0.70 | <0.001 |
| on CT | consolidation size (cm) | 0.84±0.86 | 0.73±0.79 | 1.93±0.66 | 1.91±0.78 | <0.001 |
|  | C/T ratio | 0.47±0.42 | 0.42±0.41 | 0.87±0.27 | 0.95±0.22 | <0.001 |
| Chronic pulmonary | yes | 32 | 26 | 1 | 5 | 0.023 |
| disease | no | 348 | 319 | 14 | 15 |  |
| Clinical nodal | enlarged LNs in N2 station | 339 | 318 | 12 | 9 | <0.001 |
| stage on CT | enlarged LNs in N1 station | 6 | 4 | 2 | 0 |  |
|  | normal-sized LNs | 35 | 23 | 1 | 11 |  |
| Levels of | CEA(ng/ml) | 2.87±2.69 | 2.68±2.09 | 2.28±1.48 | 6.60±6.93 | <0.001 |
| tumor markers | AFP(ng/ml) | 2.98±1.78 | 2.96±1.76 | 3.33±2.33 | 3.28±1.66 | 0.436 |
|  | CA199(U/ml) | 13.00±40.28 | 13.45±42.10 | 6.02±5.70 | 10.31±11.26 | 0.575 |
|  | CA125(U/ml) | 12.85±12.92 | 12.18±8.81 | 10.45±4.26 | 26.85±42.22 | <0.001 |
|  | CA242(U/ml) | 5.71±6.11 | 5.80±6.31 | 3.35±2.28 | 6.03±3.79 | 0.676 |
|  | CA211(ng/ml) | 1.19±0.93 | 1.16±0.87 | 1.27±0.77 | 1.68±1.83 | 0.025 |
|  | NSE(ng/ml) | 8.84±4.09 | 8.86±4.13 | 9.12±4.14 | 8.31±3.21 | 0.683 |
|  | SCC(ng/ml) | 0.85±0.83 | 0.84±0.77 | 0.78±0.33 | 1.10±1.75 | 0.255 |

RCE, routine chest examination; RCRS, respiratory or cancer related symptoms; ICD, incidental chest discovery; C/T ratio, consolidation size/ tumor size ratio

*P value acquired from one-way analysis of variance and Pearson’s chi-square tests
